# Associations between obstructive sleep apnea and dental pain and chewing discomfort in Korean adults: a nationwide cross-sectional study

**DOI:** 10.1038/s41598-023-40055-2

**Published:** 2023-08-07

**Authors:** Jae-Hyun Lee, Kyungdo Han, Su Young Lee

**Affiliations:** 1 Department of Prosthodontics and Dental Research Institute, Seoul National University School of Dentistry, Seoul, Republic of Korea; 2 Department of Statistics and Actuarial Science, Soongsil University, Seoul, Republic of Korea; 3 Department of Prosthodontics, Seoul St. Mary’s Hospital, College of Medicine, The Catholic University of Korea, 222, Banpo-daero, Seocho-gu, Seoul, 06591 Republic of Korea

**Keywords:** Health care, Dentistry, Dental public health

## Abstract

Obstructive sleep apnea (OSA) may be linked with oral health issues. This study evaluated the associations between OSA, dental pain, and chewing discomfort. Big data from a nationwide survey involving 6984 participants aged ≥ 40 years were analyzed. The STOP-Bang questionnaire was used to assess the OSA risk, categorizing the participants into low-, intermediate-, and high-risk groups. The associations of OSA risk with dental pain and chewing discomfort were evaluated using multivariate logistic regression analyses (α = 0.05). Results revealed that 50.33%, 37.50%, and 12.17% of the population belonged to the low-, intermediate-, and high-risk groups, respectively. After adjusting for covariates, a significant association emerged between OSA risk and dental pain, with adjusted odds ratios (95% confidence intervals) of 1 (reference), 1.208 (1.003–1.455), and 1.472 (1.131–1.916) for the low-, intermediate-, and high-risk groups, respectively (p = 0.0156). The adjusted odds ratio for chewing discomfort in the high-risk OSA group was 1.307 (0.977–1.748), although not significantly different from that of the low-risk group (p > 0.05). A high risk of OSA was associated with 1.472-fold increased risk of dental pain compared to those at low risk, implicating OSA as a potential risk indicator of poor oral health.

## Introduction

Sleep disorders and oral health issues are recognized as considerable public health challenges that markedly affect the quality of life of an individual^1^. Among sleep disorders, obstructive sleep apnea (OSA) impairs respiratory flow during sleep, making it a critical concern for health professionals globally^2^. OSA is not an isolated health problem; it is associated with a wide range of general health conditions^3–7^. The comprehensive implications of OSA include cerebrovascular diseases such as hypertension, stroke, and myocardial infarction^3,4,7^; metabolic disorders such as diabetes^6^; and neurological issues including depression and cognitive dysfunction^5^.

Recently, increased attention has been directed toward the interplay between oral and systemic health, sparking a new understanding of the potential impact of systemic diseases on oral health and vice versa^8–10^. This interconnectedness between oral and systemic health has broadened the scope of research and clinical practice to encompass the role of sleep disorders such as OSA in oral health outcomes^1,11,12^. A higher prevalence of periodontitis has been observed in patients with OSA than in those without OSA^11,12^. Bruxism, or nocturnal grinding and clenching of teeth, a common occurrence among adult patients with OSA, often leads to discomfort in the stomatognathic system^13^. Another crucial aspect of this discussion is temporomandibular joint disorder (TMD), which is frequently associated with OSA^14,15^. TMD can result in various manifestations, including jaw pain, facial pain, and headaches^14^. An increased prevalence of TMD symptoms has been reported among patients with OSA, suggesting a potential association between these conditions^14,15^.

Considering the close relationship between OSA and oral health issues, it is necessary to thoroughly examine these factors to better understand the intricacies of dental pain and chewing discomfort in patients with OSA. Despite the importance of this relationship, no study has investigated the unique interplay between OSA, dental pain, and chewing discomfort. To facilitate a more accessible identification of OSA, the updated STOP-Bang questionnaire, which consists of snoring, fatigue, observed apnea, high blood pressure, body mass index (BMI), age, neck circumference, and gender, was introduced^16,17^. This self-reported screening test serves as a simple and reliable means of assessing OSA risk in large populations and exhibits high sensitivity for moderate-to-severe OSA^16,17^. This study used the STOP-Bang questionnaire to decipher these associations. This cross-sectional study aimed to explore whether OSA is related to dental pain and chewing discomfort in a nationally representative population of Korean adults. The null hypothesis of the present study was that there would be no association between OSA and dental pain or chewing discomfort.

## Methods

### Study design and participants selection

This cross-sectional study analyzed nationwide data from the Korean National Health and Nutrition Examination Survey (KNHANES) conducted between 2019 and 2020 (n = 15,469). An annual survey was conducted by the Korea Disease Control and Prevention Agency to assess the general health and nutritional status of the national population. The framework for sample extraction in the KNHANES was designed based on the most recent Population and Housing Census data available at the time of the sample design, enabling representative sample extraction from the target population, which comprised all South Korean residents aged 1 year and above.

Of the participants in the 2019–2020 KNHANES, only middle-aged and older individuals aged ≥ 40 years were included. Participants with missing major variables or covariate data were excluded from the study. All participants provided informed consent for the survey, which was conducted in accordance with the Declaration of Helsinki. This survey was approved by the Institutional Review Board of the Korea Disease Control and Prevention Agency (approval numbers: 2018-01-03-C-A and 2018-01-03-2C-A). This study was conducted in compliance with the Strengthening the Reporting of Observational Studies in Epidemiology (STROBE) guidelines^18^.

### Risk of obstructive sleep apnea

This study used a modified STOP-Bang questionnaire to evaluate the risk of OSA^16,17^. The questionnaire investigated eight characteristics known to be associated with sleep apnea: Snoring, tiredness during the daytime, observed apnea, high blood pressure, BMI > 30 kg/m^2^, age > 50 years, neck circumference exceeding 36.3 cm, and being male. This study was adjusted for BMI and neck circumference to be more representative of the Asian population. BMI was set at a cut-off value of 30 kg/m^2^, which is considered indicative of severe obesity in Asians, following a specific guideline^19^. Similarly, neck circumference was set at 36.3 cm, adhering to the criteria set by the Korean Obesity Society^20^. Each affirmative response to the questionnaire was awarded one point, contributing to a maximum of eight points. Based on the accumulated points, the participants were classified into three risk levels: low risk (0–2 points), intermediate risk (3–4 points), and high risk (5–8 points)^16^. A high STOP-Bang score of 5–8 points indicated a high probability of having OSA^16^.

### Dental pain and chewing discomfort

The survey was conducted by a trained interviewer who was well-versed in the definitions of dental pain and chewing discomfort according to the guidelines provided by the KNHANES. Clear instructions and a standardized questionnaire were used to ensure that the participants understood the questions and responded consistently. The interviewer read the questions verbatim to minimize potential bias during the survey and refrained from adding comments that could have influenced the responses of the participants. Furthermore, the participants were not prompted to compare their experiences with those of others^21^.

To evaluate dental pain, participants were asked, “In the past year, have you experienced dental pain (e.g., throbbing or dull toothache or pain when consuming hot or cold food or beverages)?” Responses were recorded as either “yes” or “no.” The term “past year” refers to the year preceding the day of the survey. Dental pain was defined as any painful symptom experienced in the teeth, whether during the consumption of food or beverages. Participants who had experienced pain in the past but currently had no toothache or experienced only mild pain were also categorized as “yes.” However, in this context, dental pain excluded sensitivity symptoms, gum pain, temporomandibular joint pain, and other forms of maxillofacial pain^21^.

To evaluate chewing discomfort, the participants were asked, “Do you currently experience discomfort when chewing food due to problems in your mouth, such as with your teeth, dentures, or gums? (If you use dentures, please describe your experiences while wearing them).” The participants selected one of the following options: “severe discomfort,” “discomfort,” “moderate,” “no discomfort,” and “no discomfort at all.” The responses were dichotomized into “yes” (for those reporting “severe discomfort” or “discomfort”) and “no” (for those reporting “moderate,” “no discomfort,” or “no discomfort at all”)^21^.

### Potential confounders

Demographic, socioeconomic, and health-related variables were used as the covariates. The demographic factors included age and sex. Age was recorded both in terms of the actual years of the participants and as a dichotomous variable, with individuals aged 65 years and above categorized as older individuals^22^. Household income served as a socioeconomic variable and was categorized into low-(bottom quartile) and high-income (top three quartiles) groups. Educational attainment was classified based on whether the length of the education period was more or less than 13 years.

Health behaviors included regular smoking, drinking, and exercising. Participants who currently smoked and had smoked five or more packs during their lifetime were identified as current smokers. Heavy drinking was defined as the consumption of alcohol twice a week, with each occasion involving the intake of seven or more glasses by men and five or more glasses by women. A regular exerciser was defined as an individual who engaged in moderate-intensity exercise for at least 2 h and 30 min per week or high-intensity exercise for at least 1 h and 15 min per week.

Systemic diseases including obesity, diabetes mellitus, hypercholesterolemia, and chronic kidney disease were also assessed. Obesity was determined based on a BMI ≥ 25 kg/m^2^, according to the World Health Organization guidelines for individuals from the Asia–Pacific region^19^_._ Trained examiners measured weight, height, waist circumference, and neck circumference. The neck circumference was measured above the thyroid cartilage. Diabetes was diagnosed based on one or more of the following criteria^23^: a fasting blood glucose level of ≥ 126 mg/dL, the use of oral hypoglycemic agents or insulin injections, diagnosis by a physician, or a hemoglobin A1c level of ≥ 6.5%. Hypercholesterolemia was defined as a total plasma cholesterol level ≥ 240 mg/dL or the use of cholesterol-lowering medication^24^. Chronic kidney disease was diagnosed based on diagnosis by a physician.

### Statistical methods

Data analysis was performed using the SAS survey procedure (version 9.4; SAS Institute, Cary, NC, USA) to consider the complex sampling design of the KNHANES and provide national estimate approximations for the Korean population. General characteristics are presented as counts and percentages (standard error, s.e.m) for categorical data and as means (± s.e.m) for continuous data. The chi-square test was used to analyze categorical data, while continuous data were examined using a one-way analysis of variance. Multivariable-adjusted logistic regression analysis was conducted to investigate the odds ratios and 95% confidence intervals (CIs) for dental pain and chewing discomfort according to OSA risk. Adjustments were made in a stepwise manner: Model 1 was adjusted for demographic factors (age and sex), Model 2 included socioeconomic factors (education and income) and health behavior factors (smoking, drinking, and regular exercise), and Model 3 was further adjusted for diabetes mellitus, hypercholesterolemia, and chronic kidney disease. A subgroup analysis assessing the association between OSA risk and dental pain was conducted using Model 3, and the interaction p-values were calculated. Statistical significance was set at p < 0.05.

## Results

**Figure 1:**
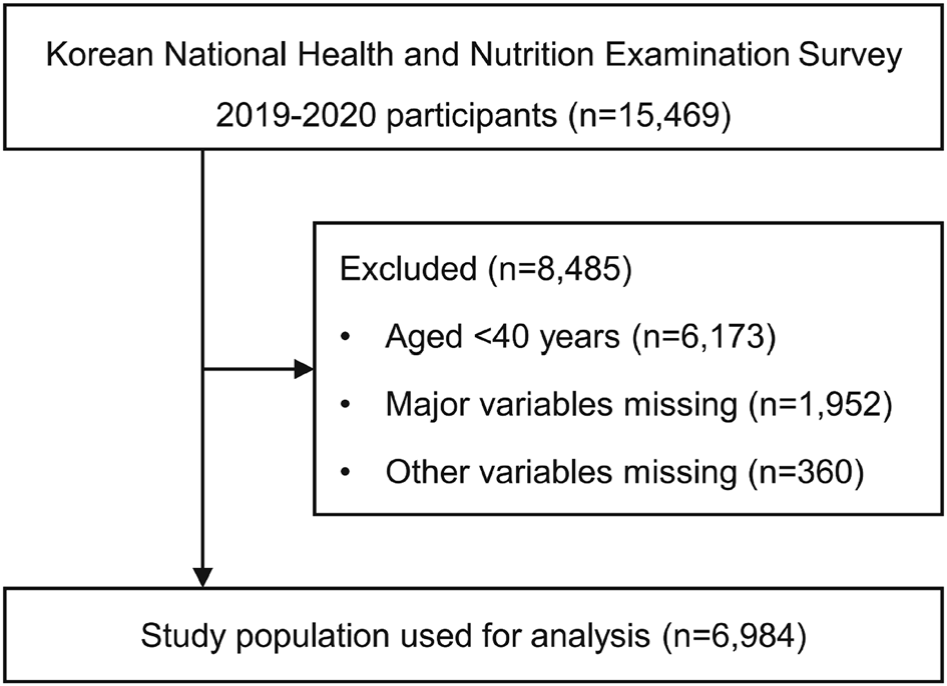
Flowchart presenting the selection of the study population.

For this study, the initial pool of participants comprised 15,469 individuals who participated in the 2019–2020 KNHANES. Those under the age of 40 years, accounting for 6173 individuals, were excluded from this analysis. Additionally, participants with incomplete data regarding major variables (1952 individuals) and covariates (360 individuals) were excluded. Consequently, a final sample of 6984 participants was obtained (Fig. 1). Table 1 shows the demographic, socioeconomic, and health-related characteristics of the study population segmented according to OSA risk. Of the 6984 participants, 3515 (50.33%) were identified as low risk, 2619 (37.50%) as intermediate risk, and 850 (12.17%) as high risk. Except for regular exercise, all confounding factors exhibited significant differences among the three groups (p < 0.01). Most participants in the high- (92.07%) and intermediate-risk groups (74.63%) were men. The weighted percentages of current smokers, heavy drinkers, and individuals with diabetes mellitus, hypercholesterolemia, and chronic kidney disease differed significantly among the three groups, with all factors being higher in the high-risk group than in the low-risk group (p < 0.0001).

**Table 1:** General characteristics of study participants according to the obstructive sleep apnea risk.

	Obstructive sleep apnea risk (STOP-Bang score)			
	Low risk	Intermediate risk	High risk	p-value
n*	3515	2619	850	
Age, years	55. 2 ± 0.28	59.05 ± 0.33	58.2 ± 0.37	< 0.0001
Age, ≥ 65 years	21.15 (0.9)	31.25 (1.24)	23.57 (1.47)	< 0.0001
Sex, men	17.23 (0.75)	74.63 (0.93)	92.07 (1.05)	< 0.0001
Education, ≥ 13 years	36.58 (1.35)	32.38 (1.48)	39.32 (2.23)	0.0014
Household income, Lowest Q1	15.75 (0.93)	19.33 (1.1)	16.46 (1.46)	0.0023
Smoking				< 0.0001
Non-	81.29 (0.75)	38 (1.16)	24.75 (1.62)	
Ex-	9.57 (0.56)	37.5 (1.12)	47.51 (1.91)	
Current	9.14 (0.66)	24.5 (1.03)	27.75 (1.73)	
Drinking				< 0.0001
Non-	35.29 (1)	26.75 (1.11)	20.9 (1.62)	
Mild to moderate	61.13 (1.02)	61.24 (1.23)	60.32 (2)	
Heavy	3.57 (0.39)	12 (0.81)	18.78 (1.57)	
Regular exercise	39.79 (0.94)	40.01 (1.14)	41.04 (1.95)	0.8351
Height, cm	159.62 ± 0.18	166.19 ± 0.22	168.82 ± 0.27	< 0.0001
Weight, kg	58.89 ± 0.18	69.01 ± 0.27	76.64 ± 0.46	< 0.0001
Waist circumference, cm	80.73 ± 0.17	88.9 ± 0.19	94.24 ± 0.35	< 0.0001
Neck circumference, cm	33.19 ± 0.05	37.07 ± 0.07	39.23 ± 0.1	< 0.0001
BMI, kg/m^2^	23.08 ± 0.06	24.95 ± 0.07	26.86 ± 0.14	< 0.0001
BMI groups				< 0.0001
< 18.5	3.99 (0.42)	1.04 (0.24)	0.17 (0.12)	
< 23	47.77 (1.02)	24.9 (1.04)	10.52 (1.19)	
< 25	23.8 (0.79)	28.41 (1.13)	23.15 (1.66)	
< 30	23.29 (0.8)	39.58 (1.15)	46.43 (2.07)	
≥ 30	1.14 (0.21)	6.08 (0.55)	19.74 (1.62)	
Diabetes mellitus	11.01 (0.64)	22.61 (0.97)	32.9 (2.02)	< 0.0001
Fasting glucose, mg/dL	99.23 ± 0.42	107.06 ± 0.58	111.73 ± 1.19	< 0.0001
Hypercholesterolemia	26.81 (0.98)	31.77 (1.06)	39.19 (1.8)	< 0.0001
Chronic kidney disease	1.71 (0.24)	4.44 (0.41)	4.44 (0.83)	< 0.0001

Data are presented as weighted % (standard error) for nominal variables and weighted mean ± standard error for continuous variables.

*BMI* body mass index.

*Unweighted n.

Table 2 presents the odds ratios (95% CIs) of dental pain and chewing discomfort according to the OSA risk. Dental pain was significantly associated with high-risk OSA in fully adjusted Model 3 (p = 0.0156). The highest odds ratio (95% CI) for dental pain, 1.472 (1.131–1.916), was found in the high-risk group in Model 3. The adjusted odds ratio for chewing discomfort in the high-risk group was 1.307 in Model 3. However, the 95% CI was 0.977–1.748, which revealed that the high-risk group was not significantly different from the low-risk group in terms of its association with chewing discomfort (p > 0.05).

**Table 2:** Odds ratios for dental pain and chewing discomfort in study participants according to obstructive sleep apnea risk scored using the STOP-Bang questionnaire.

	Obstructive sleep apnea risk	% (standard error)	Odds ratio (95% confidence interval)		
			Model 1	Model 2	Model 3
Dental pain	Low risk	23.74 (1.29)	1 (ref.)	1 (ref.)	1 (ref.)
	Intermediate risk	24.24 (1.32)	1.250 (1.037, 1.505)	1.220 (1.014, 1.467)	1.208 (1.003, 1.455)
	High risk	27.43 (2.17)	1.556 (1.199, 2.019)	1.498 (1.152, 1.949)	1.472 (1.131, 1.916)
	p-value		0.0038	0.0099	0.0156
Chewing discomfort	Low risk	19.54 (0.86)	1 (ref.)	1 (ref.)	1 (ref.)
	Intermediate risk	27.54 (1.02)	1.438 (1.199, 1.724)	1.376 (1.147, 1.650)	1.376 (1.144, 1.655)
	High risk	25.3 (1.84)	1.360 (1.029, 1.798)	1.306 (0.984, 1.735)	1.307 (0.977, 1.748)
	p-value		0.0005	0.0029	0.0034

Model 1: adjusted for age and sex; Model 2: adjusted for age, sex, education, income, smoking, drinking, and regular exercise; Model 3: adjusted for age, sex, education, income, smoking, drinking, regular exercise, diabetes mellitus, hypercholesterolemia, and chronic kidney disease.

Table 3 displays the results of the subgroup analyses and p-values for the interactions in Model 3. In all the subgroups, the odds ratios for the intermediate- and high-risk OSA groups were higher than those for the low-risk group. No significant interactions were observed in any subgroup (p > 0.05).

**Table 3:** Interactions according to subgroups concerning the association between obstructive sleep apnea and dental pain.

	Obstructive sleep apnea	% (standard error)	Adjusted odds ratio (95% confidence interval)	p for interaction
Age, years				
< 65	Low risk	24.17 (1.41)	1 (ref.)	0.757
	Intermediate risk	24.87 (1.62)	1.163 (0.917, 1.475)	
	High risk	28.90 (2.53)	1.438 (1.048, 1.972)	
≥ 65	Low risk	22.13 (1.68)	1 (ref.)	
	Intermediate risk	22.84 (1.53)	1.205 (0.918, 1.580)	
	High risk	22.66 (3.12)	1.254 (0.809, 1.944)	
Sex				
Men	Low risk	20.88 (2.41)	1 (ref.)	0.5397
	Intermediate risk	22.50 (1.38)	1.045 (0.781, 1.399)	
	High risk	27.03 (2.18)	1.273 (0.900, 1.800)	
Women	Low risk	24.33 (1.31)	1 (ref.)	
	Intermediate risk	29.35 (2.19)	1.355 (1.082, 1.696)	
	High risk	32.11 (6.39)	1.57 (0.896, 2.752)	
Household income				
Q2–Q4	Low risk	24.03 (1.43)	1 (ref.)	0.4259
	Intermediate risk	23.74 (1.43)	1.144 (0.915, 1.430)	
	High risk	26.93 (2.36)	1.379 (1.017, 1.869)	
Q1 (lowest)	Low risk	22.15 (1.96)	1 (ref.)	
	Intermediate risk	26.30 (2.36)	1.449 (1.029, 2.040)	
	High risk	29.95 (4.33)	1.878 (1.160, 3.041)	
BMI, kg/m^2^				
< 25*	Low risk	23.95 (1.34)	1 (ref.)	0.7711
	Intermediate risk	24.09 (1.54)	1.214 (0.964, 1.529)	
	High risk	28.99 (3.25)	1.641 (1.136, 2.369)	
≥ 25	Low risk	23.08 (2.07)	1 (ref.)	
	Intermediate risk	24.41 (1.67)	1.251 (0.913, 1.714)	
	High risk	26.63 (2.56)	1.44 (0.940, 2.207)	
Smoking				
Non, ex	Low risk	23.29 (1.31)	1 (ref.)	0.33
	Intermediate risk	24.38 (1.48)	1.313 (1.074, 1.606)	
	High risk	27.42 (2.52)	1.623 (1.220, 2.158)	
Current	Low risk	23.80 (2.33)	1 (ref.)	
	Intermediate risk	27.46 (3.43)	1.331 (0.924, 1.517)	
	High risk	28.18 (3.49)	1.462 (0.959, 1.656)	
Drinking				
Non, mild	Low risk	23.55 (1.30)	1 (ref.)	0.9821
	Intermediate risk	23.60 (1.33)	1.246 (1.026, 1.514)	
	High risk	26.12 (2.39)	1.535 (1.166, 2.020)	
Heavy	Low risk	28.81 (5.33)	1 (ref.)	
	Intermediate risk	28.89 (3.49)	1.271 (0.849, 1.688)	
	High risk	33.09 (4.64)	1.553 (0.936, 2.085)	
Regular exercise				
No	Low risk	23.51 (1.37)	1 (ref.)	0.8585
	Intermediate risk	23.78 (1.49)	1.192 (0.961, 1.478)	
	High risk	27.60 (2.90)	1.503 (1.066, 2.120)	
Yes	Low risk	24.08 (1.74)	1 (ref.)	
	Intermediate risk	24.92 (1.85)	1.221 (0.895, 1.666)	
	High risk	27.18 (2.82)	1.398 (0.945, 2.068)	
Diabetes mellitus				
No	Low risk	23.82 (1.34)	1 (ref.)	0.7275
	Intermediate risk	23.58 (1.43)	1.184 (0.952, 1.473)	
	High risk	26.71 (2.41)	1.457 (1.072, 1.979)	
Yes	Low risk	23.03 (2.52)	1 (ref.)	
	Intermediate risk	26.48 (2.28)	1.296 (0.826, 2.032)	
	High risk	28.90 (3.49)	1.4 (0.804, 2.438)	
Hypercholesterolemia				
No	Low risk	23.99 (1.38)	1 (ref.)	0.1912
	Intermediate risk	24.01 (1.41)	1.255 (1.006, 1.566)	
	High risk	24.71 (2.55)	1.378 (0.990, 1.917)	
Yes	Low risk	23.05 (1.83)	1 (ref.)	
	Intermediate risk	24.73 (1.96)	1.15 (0.846, 1.562)	
	High risk	31.65 (3.12)	1.568 (1.046, 2.349)	
Chronic kidney disease				
No	Low risk	23.72 (1.29)	1 (ref.)	0.6498
	Intermediate risk	24.05 (1.31)	1.177 (0.982, 1.412)	
	High risk	27.67 (2.21)	1.457 (1.119, 1.898)	
Yes	Low risk	24.57 (6.37)	1 (ref.)	
	Intermediate risk	28.15 (4.66)	1.593 (0.597, 4.249)	
	High risk	22.13 (7.39)	1.573 (0.484, 5.115)	

Odds ratios adjusted for age, sex, education, income, smoking, drinking, regular exercise, diabetes mellitus, hypercholesterolemia, and chronic kidney disease.

*BMI* body mass index, *Q1* the lowest quartile.

*The World Health Organization defines obesity for people from the Asia–Pacific region as BMI ≥ 25 kg/m^2^.

## Discussion

Dental pain was significantly associated with a high risk of OSA according to the findings of the multivariate logistic regression analysis (p = 0.0156). The high-risk OSA group had a 1.472-fold increased risk of dental pain compared with the low-risk OSA group. In contrast, for chewing discomfort, no significant difference in odds ratio was found between the low- and high-risk OSA groups after adjusting for factors such as age, sex, education, income, smoking, drinking, regular exercise, diabetes mellitus, hypercholesterolemia, and chronic kidney disease in Model 3. Consequently, the null hypothesis of the present study was partially rejected.

In the present study, the odds ratio (95% CI) of the high-risk OSA group for dental pain was 1.472 (1.131–1.916) in Model 3, which was significantly higher than that of the low-risk OSA group. Additionally, the p-values for interaction were > 0.05 in all subgroup analyses. This suggests that the association between high-risk OSA and dental pain was consistent across the different subgroups, implying an independent association between high-risk OSA and dental pain. The higher incidence of dental pain in the high-risk OSA group can be explained by the established pathophysiology of OSA^25^. Recurring instances of intermittent hypoxia, a characteristic of OSA, can potentially increase pain sensitivity and exacerbate preexisting dental pain^25^. Indeed, OSA, which is characterized by the repetitive cessation of breathing during sleep, typically induces mouth breathing, which can potentially compromise oral health^12,26^. This is likely one of the mechanisms by which OSA is associated with dental pain^26^. Symptoms associated with OSA, such as oral dryness and mouth breathing, can impede the self-cleansing capability of the oral cavity and promote the development of dental pain-related conditions such as dental caries^27^. Furthermore, OSA has been associated with parafunctional activities such as nocturnal bruxism and grinding, which could potentially cause dental pain owing to excessive forces exerted on the teeth and temporomandibular joint^13,14,28^.

Another explanation could be the fatigue experienced by patients with OSA^16,17^. Despite sleeping for extended periods, these patients often do not achieve sufficiently deep sleep^16,17^. This results in a persistent state of fatigue and sleepiness, even during the daytime, which can lead to the neglect of oral healthcare. Future studies that examine the relationship between OSA and oral health behaviors, such as brushing frequency and the use of auxiliary oral products, would be beneficial for understanding the underlying mechanisms. Additionally, several clinical anatomical indicators, such as a large, wide tongue; high, narrow palate; and reduced activity in the soft palate, are associated with OSA^29–31^_._ These structural features cause discomfort during breathing and swallowing. Simultaneously, these characteristics might hinder the appropriate use of oral hygiene instruments, such as toothbrushes, dental floss, and interdental brushes.

Based on the findings on the association between OSA risk and dental pain in the present study, an interdisciplinary approach is necessary to ensure comprehensive patient care. Patients with OSA should be referred to dental professionals and instructed to maintain appropriate oral health behaviors, including periodic professional oral examinations, to prevent dental pain. In addition, active treatment may be beneficial for some patients with severe OSA. These treatments include mandibular advancement devices^32^, oropharyngeal exercises ^33^, and orthodontic treatments, such as maxillomandibular advancement and rapid maxillary expansion^34^.

In this study, regarding the association between OSA and chewing discomfort, a statistically significant difference in odds ratios between the high- and low-risk OSA groups was present only in Model 1 (95% CI for the high-risk group, 1.029–1.798). This association, however, did not reach statistical significance in Models 2 (95% CI for the high-risk group, 0.984–1.735) and 3 (95% CI for the high-risk group, 0.977–1.748). Interestingly, in Model 3, the intermediate-risk group, with a STOP-Bang score of 3–4, still showed a significant difference from the low-risk group, which scored 0–2 (95% CI for the intermediate-risk group, 1.144–1.655). However, since a high STOP-Bang score of 5–8 is generally considered an indicator of an increased probability of OSA^16^, it is the results of the high-risk group that should be the focus of our interpretation. Hence, the association between OSA and chewing discomfort remains unclear. This is probably because chewing discomfort is a more subjective sensation than dental pain, and may be related to occlusal factors and occlusal dysesthesia^35^. In addition, chewing discomfort can be more directly influenced by oral conditions regarding dental occlusion, such as the number of remaining teeth or type of dental prostheses used^36,37^. Previous studies have reported that the use of a removable prosthesis, such as a conventional denture, is significantly related to increased discomfort compared to the use of dental implants^36,37^. However, in the present study, occlusal factors were not analyzed or controlled. To clarify the relationship between OSA and chewing discomfort, a study that controls for oral conditions such as the use of dental prostheses or the number of teeth is needed.

The strength of this study lies in its novelty in confirming the association between OSA and dental pain, emphasizing the clinical significance of dental issues in OSA, and vice versa. In addition, according to the obesity criteria proposed for the Korean population, the cut-off levels for BMI and neck circumference were changed to 30 kg/m^2^ and 36.3 cm, respectively^19,20^_._ This modified screening test for OSA, tailored to the Korean population, has been reported to have good sensitivity and specificity^38^. Furthermore, the large, representative sample size of the Korean population and statistical adjustments of a national health examination survey enhanced the reliability of the study. The large sample size, coupled with stratified multilevel probability sampling, not only improves the precision of our findings but also allows for statistical adjustments for various potential confounding factors related to OSA, enhancing the generalizability of the findings.

Despite these strengths, this study had several limitations. First, reliance on a self-report questionnaire for subjective oral health assessment may introduce bias, as individuals could either underestimate or overestimate their oral health conditions owing to various factors, including fear of treatment, unawareness, or difficulty in perceiving early signs of oral diseases. Second, owing to the large nationwide scope of the survey, the diagnosis of OSA could not be confirmed using overnight polysomnography^39,40^. Finally, the cross-sectional design of the study limited the establishment of causality between OSA and dental pain or chewing discomfort, allowing only evidence of an association. Additionally, this study did not account for other oral health factors, such as oral health behaviors, number of remaining teeth, and removable denture use. Further prospective longitudinal studies are needed to validate the findings presented in this study.

## Conclusions

Within the limitations of this cross-sectional study, a high risk of OSA was significantly associated with dental pain. The risk of experiencing dental pain in individuals at a high risk of OSA was 1.472 times greater than that in those at low risk, even after adjusting for various potential confounders, including age, sex, education, income, smoking, drinking, regular exercise, diabetes mellitus, hypercholesterolemia, and chronic kidney disease. In contrast, when examining the association between OSA risk and chewing discomfort, no significant difference was observed between the high- and low-risk OSA groups in the final adjusted model.

The insights gained from this extensive nationwide study suggest that OSA may be a potential risk marker for adverse oral health conditions, particularly dental pain. These findings emphasize the need for dental practitioners and physicians to consider an integrated approach to oral healthcare when diagnosing or treating patients with symptoms indicative of OSA. This holistic strategy could contribute to an enriched healthcare plan, ultimately aimed at enhancing both sleep quality and oral health outcomes.

## Data Availability

The datasets used and/or analyzed during the current study available from the corresponding author on reasonable request. The raw database of the Korean National Health and Nutrition Examination Survey (KNHANES) is available on the Korea Disease Control and Prevention Agency website (https://knhanes.kdca.go.kr/knhanes).
